# Modeling and Simulations of Buongiorno’s Model for Nanofluid in a Microchannel with Electro-Osmotic Effects and an Exothermal Chemical Reaction

**DOI:** 10.3390/nano11040905

**Published:** 2021-04-01

**Authors:** Ammarah Raees, Muhammad Raees-ul-Haq, Muavia Mansoor

**Affiliations:** 1 Faculty of Computer Science and Software Engineering, Huaiyin Institute of Technology, Huaian 223003, China; raees@hyit.edu.cn; 2 Department of Mathematics, University of Wah, Quaid Aveneu, Wah Cantt, Northern Punjab Rawalpindi 47040, Pakistan; muavia.mansoor@uow.edu.pk

**Keywords:** electroosmotic effects, Arrhenius kinetics, nanofluids, Buongiorno’s model, slip boundary conditions, MHD

## Abstract

The article presents a mathematical model for the magnetized nanofluid flow and heat transfer with an exothermic chemical reaction controlled by Arrhenius kinetics. Buongiorno’s model with passive boundary condition is employed to formulate the governing equation for nanoparticles concentration. The momentum equation with slip boundary conditions is modelled with the inclusion of electroosmotic effects which remain inattentive in the study of microchannel flows with electric double layer (EDL) effects. Conclusions are based on graphical and numerical results for the dimensionless numbers representing the features of heat transfer and fluid flow. Frank-Kamenetskii parameter resulting from the chemical reaction showed significant effects on the optimization of heat transfer, leading to increased heat exchangers’ effectiveness. The Hartmann number and slip parameter significantly affect skin friction, demonstrating the notable effects of electroosmotic flow and the exothermic chemical reaction on heat transfer in microchannels. This analysis contributes to prognosticating the convective heat transfer of nanofluids on a micro-scale for accomplishing successful thermal designs.

## 1. Introduction

Fluid flow and heat transfer in the microchannel sufficiently occupy a dominant role in several potential applications which typically include microdevices [1,2] based on the microfabrication techniques, micro-electro-mechanical systems (MEMS) [3,4], biomedical reaction chambers, and heat exchangers for electronics cooling. In this specific context, many experimental and analytical studies are conducted to interpret the essential properties of both heat transfer and fluid motion in both micro and nano-scale based on various parametric effects. In the micro-scale system, the fluid motion at the fluid-solid interface is profoundly influenced by several fundamental parameters like viscosity, slip-condition, electric double layer (EDL), electroosmosis, zeta potential and so forth [5,6,7,8,9,10,11,12,13,14]. Consequently, it is substantial to adequately investigate these necessary parameters, which provide essential estimating tools for heat transfer in microdevices.

A considerable number of papers focused on the microflows has described the dependence of flow length scale and surface attributes on the boundary conditions at the channel plates. The investigation showed the slip effect [15] can occur due to the microscopic roughness [16] of the surface or by Hydrophobic smooth surfaces [17,18,19] such as in polydimethylsiloxane (PDMS) materials can make channels or hydrophobic liquids. Its also been described that the slip conditions occurred at the microchannel walls for both Newtonian [20,21] and Non-Newtonian fluids [15,16,17,18,19]. Bhagavatula and Castro [22] derived the mathematical model for describing the rheological behavior of the coated substance for the high shear rates based on the Carreau viscosity model with the linear Navier slip boundary conditions. Babaie et al. [23] investigated the thermal transport attributes of the electroosmotic pressure-driven flow of power-law fluids through a slit microchannel. They reasonably concluded that the thermic behavior of the flowing fluid depended on the non-dimensional Debye-Huckel parameter, the velocity scale ratio, and the Joule heating parameter. Shojaeian and Kosar [24] analytically analyzed the entropy generation and convective heat transfer in both Newtonian and non-Newtonian fluid attributed to the slip boundary conditions. They found that the slip coefficient is one of the parameters which greatly influence the heat transfer characteristics in the Newtonian and non-Newtonian fluid. You and Guo [25] incorporated the electric current density balance (ECDB) mode and Navier slip boundary conditions to examine the impacts of EDL and performed the stability analysis. They observed that the cause of the destabilization by the potential EDL effect on the fluid flow and its mean velocity is greatly stabilized by the boundary slip parameter.

Currently, many investigative researchers have computed the convective heat and mass transfer of the nanofluids in the microchannel with the EDL effects, the possible inclusion of slip effects, magnetohydrodynamics (MHD), viscous dissipation and so on. Xie and Jian [26] performed the entropy generation to study the MHD electroosmotic two-layer flow in the microchannel and proved the strong dependence of entropy generation on both the fluid velocity and temperature fields. Jiang et al. [27] theoretically investigated the overlapping EDL induced-electroviscous effect (EVE) on the microchannel flow by considering both the no-slip condition and slip condition. Zhao et al. [28] utilized Buongiorno’s model [29] to adequately investigate the EDL effects on the nanofluid flow through the microchannel. They also examined [30] the potential effect of viscous dissipation and MHD with the EDL effect on the nanofluid flow through microchannel by properly using the passively controlled nanofluid model [31]. Based on their analysis they found that EDL occupies a primary role in nanoparticles volumetric distribution and positively affects the heat transfer rate of nanofluid. Raees et al. [32] numerically examined the heat and mass transfer phenomenon in the microchannel under electrokinetic effects using sinusoidal boundary conditions. Niazi and Xu [33] have accurately modeled and provided the entropy analysis of the two-layer MHD nanofluid flow in the microchannel considering electroosmotic effects by employing Buongiorno’s model.

One of the essential features that typically provides heat to the fluid efficiently is due to the exothermic chemical reaction on the surface. This chemical reaction is represented by a first-order kinetic equation known as Arrhenius kinetics. The Arrhenius equation is a relevant entity to determine the rate at which a chemical system changes its temperature as a result of a chemical reaction. This relationship links the rate of reaction and the temperature for many physical systems. The most popular form of the Arrhenius equation [34] is given by:

(1) $$A\to B,\;\mathrm{rate}=k_{0}a\exp\left(-\frac{E}{RT}\right),$$

where *A* and *B* are the reactants, $k_{0}$, *a*, *E* and *R* denotes the pre-exponential factor, the reactant $A^{\prime }s$ concentration, activation energy and fluid constant, respectively. Significant studies have been done by many investigators [35,36,37,38,39,40]. Merkin and Mahmood [35] devised a model to investigate the reactive component in a fluid-saturated porous medium. They have obtained multiple results for this problem and concluded the stability of the upper and lower branch solutions. Chaudhary et al. [36] presented the disjoint bifurcation diagrams to discuss the critical ambient temperature at the surface containing catalytic reaction in a forward stagnation flow. Merkin and Pop [37] examined the effect of Arrhenius kinetics in a stagnation flow with shrinking/stretching surface. They obtained three-branch solutions and discussed the location of critical points and hysteresis bifurcation for non-dimensional temperature and concentration profiles. Zhang et al. [38] considered the three types of nanoparticles (Cu, Al2O3, Ag) and investigated there influence in a fluid flow on a horizontal plane in a porous medium. They have additionally included the effects of chemical reaction, MHD and radiation. Subsequently, they graphically analyzed the impact of these three nanoparticles with several physical parameters arising in the problem. Rahman [39] numerically analyzed the heat transfer in a tilted nanofluid porous square cavity in the presence of Arrhenius kinetics and MHD. They concluded the Frank-Kamenetskii and Rayleigh numbers played a vital role in heat generation. Reference [40] investigated the impact of chemical reactions on heat and mass transfer phenomenon in the nanofluid flow in a triangular cavity. He observed the rise in the Rayleigh number decreased the Nusselt number while increasing the Frank-Kamenetskii number enhances the rate of heat transfer.

It is apparent from the previous studies that slight attention is directed to investigate the effect of exothermic chemical reactions in the microchannel particularly in the presence of electrokinetic effects. Therefore, in this paper, we have examined the flow and heat transfer characteristics of a nanofluid in a microchannel with MHD field effects, EDL, viscous dissipation effects and chemical reaction incorporating the slip boundary conditions. We have employed Buongiorno’s model with the passive boundary conditions. As such, to the author’s best knowledge, this particular problem involving the EDL and exothermic chemical reaction in a microchannel has been unreported yet in the literature. The numerical solutions are obtained, and the physical analysis is performed on these solutions to interpret the phenomenon of fluid motion and heat transfer w.r.t several parameters of physical importance in the problem.

## 2. Governing Equations

### Theory and Mathematical Modelling

**Figure 1:**
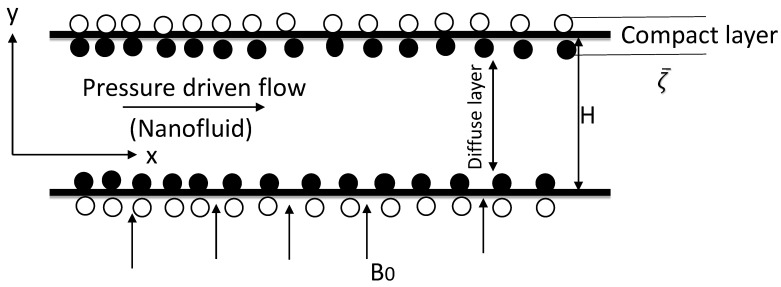
A schematic of the geometry.

We consider a pressure-driven, steady, and laminar nanofluid flow through a microchannel with the EDL effects and an exothermal chemical reaction as shown in Figure 1. While the magnetic field is sought externally. The temperature at the bottom plate of the microchannel is kept at $T_{1}$ while at the top plate it is taken as $T_{2}$. In the fluid flow at macroscale, the no-slip boundary condition is plausible because the potential slip-boundary condition is relatively insignificant. But at a microscale level, the influence of slip boundary on fluid flow is noticeable and needs to be adequately taken into account. Consequently, the slip boundary condition is employed with the Navier hypothesis [25]

(2) $$u_{\mathrm{wall}}=\beta^{*}{\left.\frac{du}{dy}\right|}_{\mathrm{wall}},$$

here $\beta^{*}$ denotes the slip parameter which gives the characteristic slip length.

Buongiorno’s nanofluid model is properly utilized for the mathematical formulation of the specific problem. The passive condition is applied at the upper wall boundary while the concentration of nanoparticles at the lower wall remains constant $C_{1}$. The magnetic field having strength B is applied across the *y*-axis. The length of the microchannel is denoted by *L* and *H* represents the distance between the two walls of the microchannel. The physical model with the corresponding boundary conditions. Scientifically based on the earlier considerations, the non-dimensional mathematical model [29], ref. [26] is written as follows

(3) $$\begin{array}{ccc} & & \nabla^{2}\psi =-\frac{\rho_{e}}{\varepsilon_{0}\varepsilon_{r}}, \end{array}$$

(4) $$\begin{array}{ccc} & & \nabla \cdot V=0, \end{array}$$

(5) $$\begin{array}{ccc} & & \rho \left(V\cdot \nabla \right)V=-\nabla p+\mu \nabla^{2}V+F, \end{array}$$

(6) $$\begin{array}{ccc} & & \left(V\cdot \nabla \right)T=\alpha \nabla^{2}T+\tau \left[D_{B}\nabla T\;\nabla C+\left(\frac{D_{T}}{T_{0}}\right)\nabla T\;\nabla T\right]\\ & & \;\;+\frac{\mu }{\rho c_{p}}\Phi +Qk_{0}a\exp\left(-E/RT\right), \end{array}$$

(7) $$\begin{array}{ccc} & & \left(V\cdot \nabla \right)C=D_{B}\nabla^{2}C+\left(\frac{D_{T}}{T_{0}}\right)\nabla^{2}T, \end{array}$$

where ψ denotes the electric potential caused by EDL effects, $\varepsilon_{0}$ represents the permittivity of vacuum, the fluid’s dielectric constant and the electrical conductivity is given by $\varepsilon_{r}$ and σ respectively, the velocity vector and the charge density is represented by V and $\rho_{e}$ respectively, $F=\rho_{e}E_{e}+J\times B$ represents the body force which constitutes the both electroosmosis and electromagnetic forces, $E_{e}$ denotes the electric field strength caused by the EDL, J=σ(E+V×B) is the ion current density, E and B is the electrical and imposed magnetic field respectively, *p* denotes the pressure, ρ, μ and α represents the fluid density, the dynamic viscosity and the thermal diffusivity respectively, *T* denotes the temperature, $T_{0}$ denotes the reference temperature, Φ gives the viscous dissipation, *Q* is the exothermicity factor, *a* gives the concentration of reactant A, *E* denotes the activation energy, *R* is a fluid constant, $k_{0}$ is a pre-exponential factor, the volume fraction of nanoparticles is represented by *C*, $\tau =\frac{{\left(\rho c_{p}\right)}_{p}}{{\left(\rho c_{p}\right)}_{f}}$ is the heat capacity ratio and the subscripts *f* and *p* denotes the difference in quantities for fluid and solid particles, $D_{T}$ and $D_{B}$ represent the thermophoretic and the Brownian diffusion coefficients respetively. The following assumptions are taken in account for the analysis

- The electric force $E_{e}\rho_{e}$ is due to the EDL and the electric field strength is directed towards x-axis (i.e., $E_{e}=\left(E_{x},0\right)$) because the freely charged particles on surface of EDL will alter along the direction of fluid flow.
- The fluid flow in the microchannel is assumed parallel to the x-axis. So only the velocity component of fluid along the x-axis is non-zero.
- The induced magnetic field is neglected because the minimal magnetic Reynolds number (<<1) is considered. While the imposed magnetic field based on the Ohm’s law and these assumptions can be written as $B=(0,B_{0})$.
- The change in temperature and nanparticles volume fraction are kept constant along horizontal direction (i.e., $\frac{\partial T}{\partial x}=\frac{\partial C}{\partial x}=0$ ).
- The applied electric field is neglected and the fluid motion is pressure driven, that is, E=0.

Keeping in view all the above assumptions, the reduced dimensional form of Equations (3)–(7) can be written as follows:

(8) $$\begin{array}{ccc} & & \frac{d^{2}\psi }{dy^{2}}=-\frac{\rho_{e}}{\varepsilon_{0}\varepsilon_{r}}, \end{array}$$

(9) $$\begin{array}{ccc} & & \mu \frac{\partial^{2}u}{\partial y^{2}}-\frac{\partial p}{\partial x}+E_{x}\rho_{e}-\sigma B_{0}^{2}u=0, \end{array}$$

(10) $$\begin{array}{ccc} & & \alpha \left(\frac{\partial^{2}T}{\partial y^{2}}\right)+\tau \left[D_{B}\frac{\partial T}{\partial y}\frac{\partial C}{\partial y}+\left(\frac{D_{T}}{T_{0}}\right){\left(\frac{\partial T}{\partial y}\right)}^{2}\right]+\frac{\mu }{\rho c_{p}}{\left(\frac{du}{dy}\right)}^{2}\\ & & +Qk_{0}a\exp\left(-E/RT\right)=0, \end{array}$$

(11) $$\begin{array}{ccc} & & D_{B}\frac{\partial^{2}C}{\partial y^{2}}+\left(\frac{D_{T}}{T_{0}}\right)\frac{\partial^{2}T}{\partial y^{2}}=0. \end{array}$$

For this problem, the slip boundary conditions are employed for the velocity while the temperature boundary conditions are kept isothermal. The pertinent detail is associated to the passive boundary condition which is employed at the upper wall of the microchannel to describe the nanoparticle concentration. Moreover, zeta potential (ζ) is considered within the diffusive and the compact layer. Hence, the associated boundary conditions for Equations (8)–(11) are given as

(12) $$\begin{array}{ccc} & & \psi \left(p\right)=\bar{\zeta },\;u=\beta^{*}\frac{du}{dy},\;T\left(y\right)=T_{1},\;C\left(y\right)=C_{1}\;\;\;\;\;\;\;\;\;at\;y=0,\\ & & \psi \left(y\right)=\bar{\zeta },\;u=-\beta^{*}\frac{du}{dy},\;T\left(y\right)=T_{2},\;D_{B}\frac{\partial C}{\partial y}+\left(\frac{D_{T}}{T_{0}}\right)\frac{\partial T}{\partial y}=0\;\;at\;y=H, \end{array}$$

where $\bar{\zeta }=ze_{0}\zeta /\left(k_{b}\hat{T}\right)$. For the uniform dielectric constant, the equilibrium Boltzmann distribution can be defined as

(13) $$\rho_{e}=-2n_{0}\hat{z}e\sinh\left(\frac{\hat{z}e\psi }{k_{b}\hat{T}}\right),$$

where $n_{0}$, $\hat{z}$, *e*, $k_{b}$ and $\hat{T}$ represent, the bulk ionic concentration, the valence of ions, the fundamental charge, the Boltzmann constant and the absolute temperature respectively. As per the linear approximation proposed by Debye-Hückel, $|\hat{z}e\psi |<|k_{b}\hat{T}|$, the non-dimensional Poisson-Boltzmann equation can be written as

(14) $$\begin{array}{ccc} & & \frac{d^{2}\psi }{dy^{2}}-\left(\frac{2n_{0}{\hat{z}}^{2}e^{2}}{\varepsilon_{0}\varepsilon_{r}k_{b}\hat{T}}\right)\psi =0. \end{array}$$

Now transforming the dimensional variables into the non-dimensional ones by using the following similarity variables

(15) $$\begin{array}{ccc} & & \eta =\frac{y}{H},\;\Psi \left(\eta \right)=\frac{ze_{0}\psi }{k_{b}\hat{T}},\;U\left(\eta \right)=\frac{u(y)}{U_{m}},\;\theta \left(\eta \right)=\frac{E(T-T_{0})}{RT_{0}^{2}},\\ & & \phi \left(\eta \right)=\frac{C-C_{0}}{C_{1}-C_{0}}. \end{array}$$

Substituting the above mention similarity transformations into Equations (8)–(11) and (12), we obtain the following equations:

(16) $$\begin{array}{ccc} & & \Psi^{\prime \prime }-\kappa^{2}\Psi =0, \end{array}$$

(17) $$\begin{array}{ccc} & & U^{\prime \prime }\left(\eta \right)+P-Ha^{2}U\left(\eta \right)+\kappa^{2}\Psi \left(\eta \right)=0, \end{array}$$

(18) $$\begin{array}{ccc} & & \theta^{\prime \prime }\left(\eta \right)+N_{b}\theta^{\prime }\left(\eta \right)\phi^{\prime }\left(\eta \right)+N_{t}\theta^{\prime }{\left(\eta \right)}^{2}+F_{k}\exp\left(\theta \right)+BrU^{\prime }{\left(\eta \right)}^{2}=0, \end{array}$$

(19) $$\begin{array}{ccc} & & \phi^{\prime \prime }\left(\eta \right)+\frac{N_{t}}{N_{b}}\theta^{\prime \prime }\left(\eta \right)=0, \end{array}$$

with the associated boundary conditions written as

(20) $$\begin{array}{ccc} & & \Psi \left(0\right)=\zeta ,\;U\left(0\right)=\beta U^{\prime }\left(0\right),\;\theta \left(0\right)=\delta_{\theta_{1}},\;\phi \left(0\right)=1,\;\\ & & \Psi \left(1\right)=\zeta ,\;U\left(1\right)=-\beta U^{\prime }\left(1\right),\;\theta \left(1\right)=\delta_{\theta_{2}},\;N_{b}\phi^{\prime }\left(1\right)+N_{t}\theta^{\prime }\left(1\right)=0, \end{array}$$

where $\beta =\beta^{*}/H$ denotes the dimensionless coefficient of Navier-slip boundary condition, $\delta_{\theta_{1}}=\frac{E(T_{1}-T_{0})}{RT_{0}^{2}}$ and $\delta_{\theta_{2}}=\frac{E(T_{2}-T_{0})}{RT_{0}^{2}}$ are constants, κ, Ha, *P*, $N_{b}$, $N_{t}$, $F_{k}$ and Br are, respectively, the electro-osmotic parameters, the Hartmann number, the constant pressure, the Brownian motion parameter, the thermophoresis parameter, the Frank-Kamenetskii number and the Brinkman number, which are defined as follows:

(21) $$\begin{array}{ccc} & & \kappa =\hat{z}eH\sqrt{\frac{2n_{0}}{\varepsilon_{0}\varepsilon_{r}k_{b}\hat{T}}},\;Ha=B_{0}H\sqrt{\frac{\sigma }{\mu }},\;P=-\frac{H^{2}}{\mu U_{m}}\frac{dp}{dx},\;U_{m}=\frac{\varepsilon_{r}E_{x}k_{b}\hat{T}}{\mu e\hat{z}}\\ & & N_{b}=\frac{\tau D_{B}\left(C_{1}-C_{0}\right)}{\alpha },\;N_{t}=\frac{\tau D_{T}RT_{0}}{\alpha E},\;F_{k}=\frac{Qk_{0}Ee^{\left(E/RT_{0}\right)}H^{2}}{\alpha RT_{0}^{2}},\;Br=Pr.Ec, \end{array}$$

where Pr=ν/α denotes the Prandtl and $Ec=\left(U_{m}^{2}E\right)/c_{p}RT_{0}^{2}$ represents the Eckert number, respectively. To further investigate the heat and mass transfer phenomenon in a microchannel, we define the physical quantities that is, the skin friction ($C_{f1}$, $C_{f2}$), the Nusselt ($Nu_{1}$, $Nu_{2}$) and the Sherwood numbers ($Sh_{1}$, $Sh_{2}$) are as follows:

(22) $$\begin{array}{ccc} & & C_{f1}=\frac{\tau_{w1}}{\frac{1}{2}\rho U_{m}^{2}},\;C_{f2}=\frac{\tau_{w2}}{\frac{1}{2}\rho U_{m}^{2}},\;Nu_{1}=\frac{HEq_{T1}}{kRT_{0}^{2}},\;Nu_{2}=\frac{HEq_{T2}}{kRT_{0}^{2}},\\ & & Sh_{1}=\frac{Hq_{C1}}{D_{B}\left(C_{1}-C_{0}\right)},\;Sh_{2}=\frac{Hq_{C2}}{D_{B}\left(C_{1}-C_{0}\right)}, \end{array}$$

where subscripts 1,2 represents the lower and upper walls shear stress, heat flux and mass flux denoted as $\tau_{w1}$, $\tau_{w2}$, $q_{T1}$, $q_{T2}$, $q_{C1}$ and $q_{C2}$ and defined as follows:

(23) $$\begin{array}{ccc} & & \tau_{w1}=\mu {\left.\frac{du}{dy}\right|}_{y=0},\;\tau_{w2}=\mu {\left.\frac{du}{dy}\right|}_{y=H},\;q_{T1}=-k{\left.\frac{dT}{dy}\right|}_{y=0},\;q_{T2}=-k{\left.\frac{dT}{dy}\right|}_{y=H}\\ & & q_{C1}=-D_{B}{\left.\frac{dC}{dy}\right|}_{y=0},\;q_{C2}=-D_{B}{\left.\frac{dC}{dy}\right|}_{y=H} \end{array}$$

using Equations (15) and (23) into Equation (22), we obtain

(24) $$\begin{array}{ccc} & & ReC_{f1}=2U^{\prime }\left(0\right),\;ReC_{f2}=2U^{\prime }\left(1\right),\;Nu_{1}=-\theta^{\prime }\left(0\right),\;Nu_{2}=-\theta^{\prime }\left(1\right)\\ & & Sh_{1}=-\phi^{\prime }\left(0\right),\;Sh_{2}=-\phi^{\prime }\left(1\right), \end{array}$$

where the Reynolds number, Re, is gived by:

$$Re=\frac{HU_{m}}{\nu }.$$

## 3. Results and Discussion

### 3.1. Solutions & Simulations

Equations (16) and (17) are both linear second-order ordinary differential equations(ODE), hence these equations are solved for their exact solutions. MATLAB built-in function dsolve is used to solve Equations (16) and (17) with boundary conditions Equation (20) for ψ and *U*. Following are the expressions for the exact solutions of ψ and *U*.

(25) $$\psi \left(\eta \right)=\frac{\zeta e^{-\eta \kappa }\left(e^{2\eta \kappa }+e^{\kappa }\right)}{e^{\kappa }+1}$$

(26) $$\begin{array}{cc} u\left(\eta \right)= & \frac{1}{{\mathrm{Ha}}^{2}\left(e^{\kappa }+1\right)\left(\beta \left(-\mathrm{Ha}\right)+e^{\mathrm{Ha}}\left(\beta \mathrm{Ha}+1\right)+1\right)\left(\mathrm{Ha}-\kappa \right)\left(\mathrm{Ha}+\kappa \right)}\times \\ \; & e^{-\eta (2\mathrm{Ha}+\kappa )}[\zeta {\mathrm{Ha}}^{2}\kappa^{2}\left(\beta \mathrm{Ha}-1\right)e^{2\eta (\mathrm{Ha}+\kappa )}+\zeta {\mathrm{Ha}}^{2}\kappa^{2}\left(\beta \mathrm{Ha}-1\right)e^{2\eta \mathrm{Ha}+\kappa }-\\ \; & \zeta {\mathrm{Ha}}^{2}\kappa^{2}\left(\beta \mathrm{Ha}+1\right)e^{2\eta \mathrm{Ha}+\mathrm{Ha}+\kappa }-\zeta {\mathrm{Ha}}^{2}\kappa^{2}\left(\beta \mathrm{Ha}+1\right)e^{2\eta \kappa +2\eta \mathrm{Ha}+\mathrm{Ha}}+\\ \; & e^{\eta (3\mathrm{Ha}+\kappa )}\left(\kappa^{2}P-{\mathrm{Ha}}^{2}\left(\zeta \kappa^{2}\left(\beta \kappa -1\right)+P\right)\right)+\\ \; & e^{\eta (\mathrm{Ha}+\kappa )+\mathrm{Ha}}\left(\kappa^{2}P-{\mathrm{Ha}}^{2}\left(\zeta \kappa^{2}\left(\beta \kappa -1\right)+P\right)\right)+\\ \; & e^{\left(\eta +1)(\mathrm{Ha}+\kappa \right)}\left({\mathrm{Ha}}^{2}\left(\zeta \kappa^{2}\left(\beta \kappa +1\right)-P\right)+\kappa^{2}P\right)+\\ \; & e^{\eta \kappa +3\eta \mathrm{Ha}+\kappa }\left({\mathrm{Ha}}^{2}\left(\zeta \kappa^{2}\left(\beta \kappa +1\right)-P\right)+\kappa^{2}P\right)-P\left(\beta \mathrm{Ha}-1\right)\left({\mathrm{Ha}}^{2}-\kappa^{2}\right)e^{\eta (2\mathrm{Ha}+\kappa )}-\\ \; & P\left(\beta \mathrm{Ha}-1\right)\left({\mathrm{Ha}}^{2}-\kappa^{2}\right)e^{\eta \kappa +2\eta \mathrm{Ha}+\kappa }+P\left(\beta \mathrm{Ha}+1\right)\left({\mathrm{Ha}}^{2}-\kappa^{2}\right)e^{\eta \kappa +2\eta \mathrm{Ha}+\mathrm{Ha}}+\\ \; & P\left(\beta \mathrm{Ha}+1\right)\left({\mathrm{Ha}}^{2}-\kappa^{2}\right)e^{\eta \kappa +2\eta \mathrm{Ha}+\mathrm{Ha}+\kappa .}] \end{array}$$

#### Numerical Procedure

Equations (18) and (19) are non-linear ODEs, hence it is difficult to solve for their exact solutions, therefore a numerical method is adopted to perform numerical simulations as described in [41]. The non-linearity of equations are dealt by employed the Picard’s method. Mathematically, the Picard’s algorithm for Equations (18) and (19) is described as follows.

The selected computational domain is [0,1] and every point is denoted by its position $(\eta^{i}$, where the superscript *i* indicates the cell position. The central difference scheme is emplyed for spatial discretization. The uniform mesh size for η is given by Δη, while $\eta_{i}=\eta_{0}+i\Delta \eta$. Denote the numerical solution by $\theta_{i}^{n}=\theta^{n}\left(\eta_{i}\right)$ and $\phi_{i}^{l}=\phi^{l}\left(\eta_{i}\right)$, where *n* represents the iteration index. For Equations (18) and (19), the discretized equations are developed with accuracy order $O(\Delta \eta^{2})$.

For the convergence of iterative procedure, $10^{-6}$ is selected as a tolerance error for all field variables. The iterative scheme is stopped with the below mentioned convergence condition:

(27) $$\frac{\sum_{i=1}^{N}\left|\Phi_{i}^{n+1}-\Phi_{i}^{n}\right|}{\sum_{i=1}^{N}\left|\Phi_{i}^{n+1}\right|}\leq 10^{-6},\;\;n\geq 1,$$

where Φ denotes the dependent variables θ or ϕ, while *N* is the total grid points along η-direction, and *n* is the iteration index.

### 3.2. Grid Independence Test

**Figure 2:**
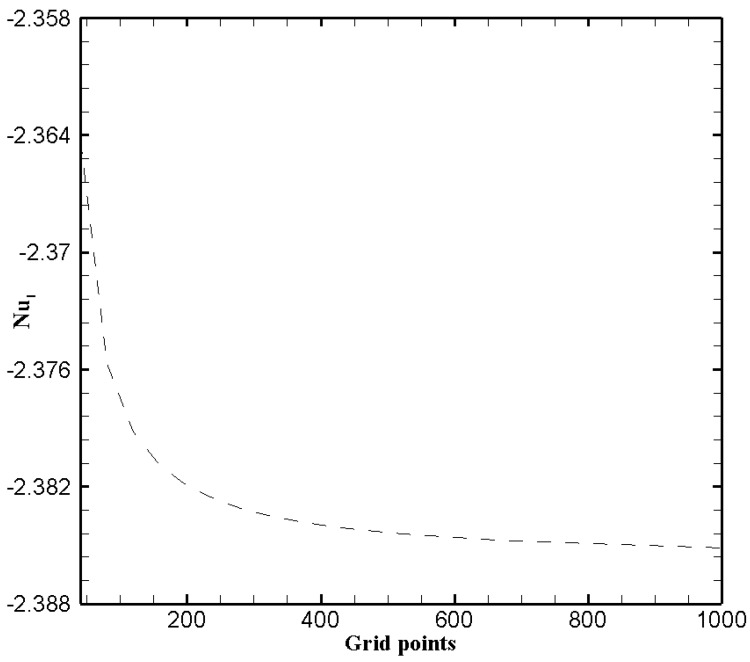
Grid independence test.

To further verify the results obtained from numerical scheme, it is imperative to conduct a grid sensitivity investigation on the obtained results. Thus twenty five different number of grid sizes varying from 40 to 1000 points are tested. The numerical simulations for grid sensitivity analysis is performed for particular values of the various parameters κ=ζ=1, $H_{a}=P=1$, β=0.05, $B_{r}=1$, $N_{b}=0.2,N_{t}=0.1$, $\delta_{\theta 1}=1.2$ and $F_{k}=1$. The test is performed by giving the graphical representation of the local Nusselt number on the left wall w.r.t. the grid size. We see from the Figure 2, the numeric Nusselt number becomes independent of the number grid points when they are greater than 400. Hence, a total number of 400 grid points is considered to be suitable for the remaining analysis.

### 3.3. Effects of Hartmann Number (Ha) and Slip Parameter (β)

**Figure 3:**
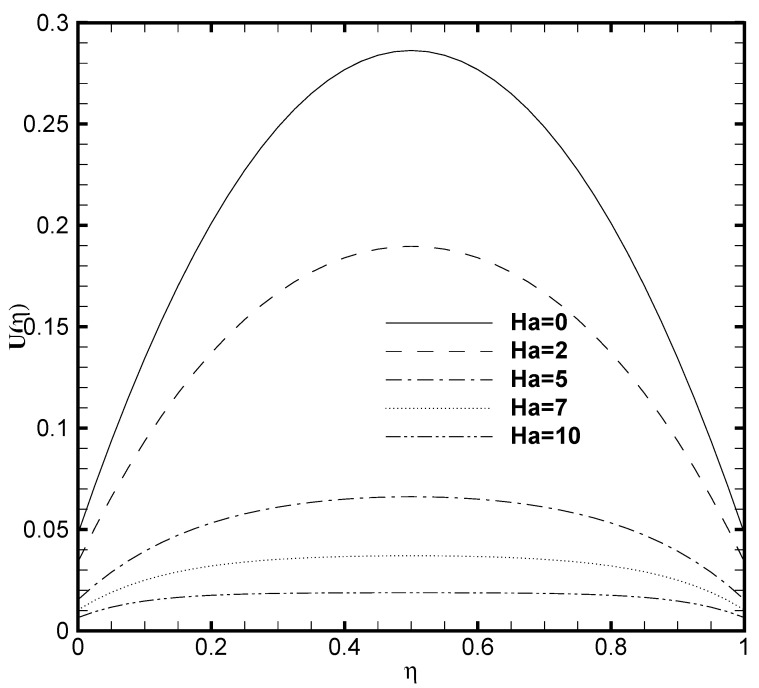
Velocity profile U(η) for several values of Hartmann number Ha when ζ=P=κ=1 and β=0.05.

**Figure 4:**
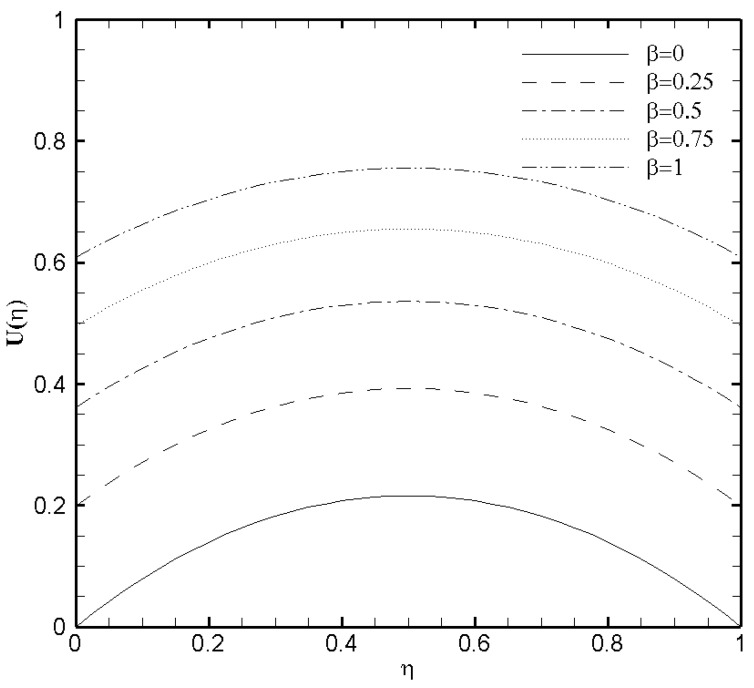
Velocity profile U(η) for several values of slip parameter β when ζ=P=κ=1 and Ha=1.

Figure 3 presents the profound influence of Hartmann number Ha on the velocity profile U(η). It can be carefully observed that the increased value of Ha depreciates the flow velocity. The velocity distribution curve in common is a parabola in the specific case of Ha=0, which corresponds respectively to the absence of magnetic field. But as Ha≫1, the velocity profile typically gets flatten at the middle of the microchannel because the more considerable values of the magnetic field decreases the flow velocity and hinders the likely formation of turbulence. The velocity distribution in the microchannel with the variation of slip parameter β is plotted in Figure 4. The combined effect of the EDL and increasing value of boundary slip causes the possible enhancement of the velocity curve. The reasons is, the slip velocity, $U_{wall}$, at the boundary is proportional to $\frac{dU_{wall}}{d\eta }$. The $\frac{dU_{wall}}{d\eta }$ value approaches zero near the wall because of the evident effect of EDL. It can equally be seen that for the non-slip condition (β=0) on both the upper and lower wall, a symmetric profile for velocity is observed at the channel center. Basically, due to the considerable influence of the EDL in the microchannel flow weakens the effect of boundary slip.

### 3.4. Effects of Brinkmann Number (Br) and Frank-Kamenetskii Number ($F_{k}$)

**Figure 5:**
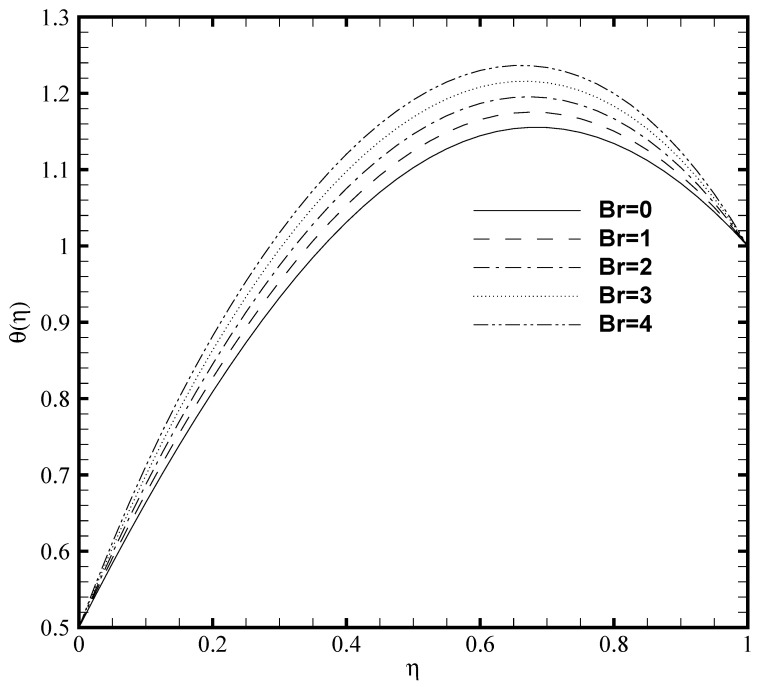
Temperature profile θ(η) for several values of Brinkmann number Br when $\zeta =P=\kappa =Ha=\delta_{\theta 2}=F_{k}=1$, $N_{b}=0.2$, $N_{t}=0.1$, β=0.05 and $\delta_{\theta 1}=0.5$.

**Figure 6:**
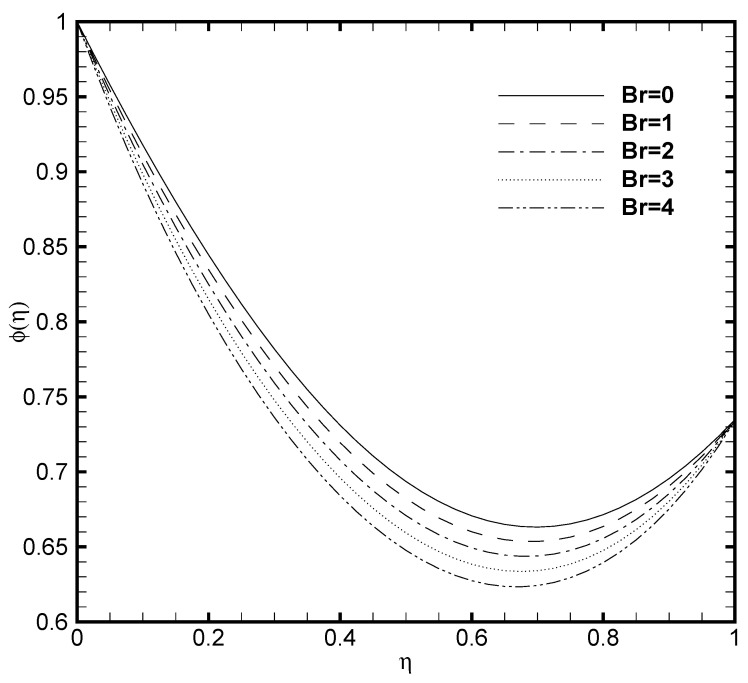
Nanoparticles concentration profile ϕ(η) for several values of Brinkmann number Br when $\zeta =P=\kappa =Ha=\delta_{\theta 2}=F_{k}=1$, $N_{b}=0.2$, $N_{t}=0.1$, β=0.05 and $\delta_{\theta 1}=0.5$.

**Figure 7:**
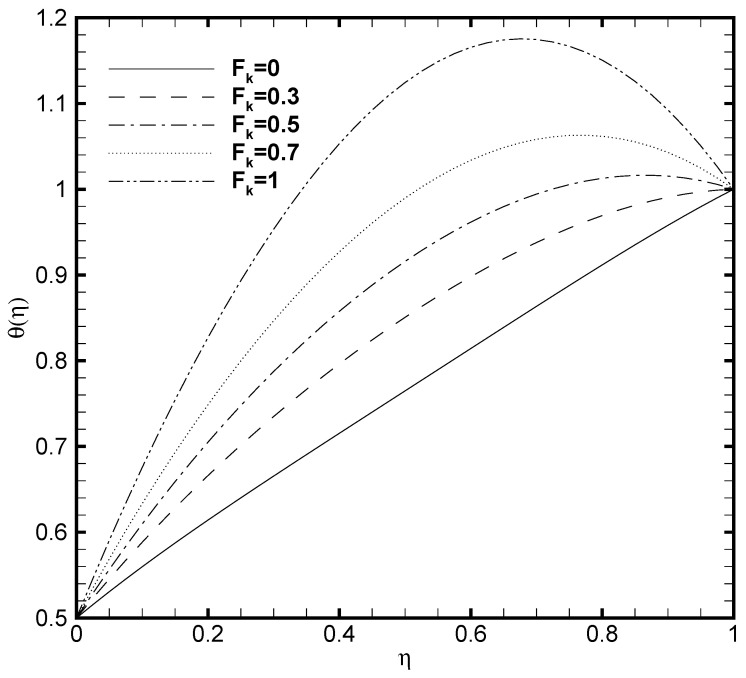
Temperature profile θ(η) for several values of Frank-Kamenetskii number $F_{k}$ when $\zeta =P=\kappa =Ha=Br=\delta_{\theta 2}=1$, $N_{b}=0.2$, $N_{t}=0.1$, β=0.05 and $\delta_{\theta 1}=0.5$.

**Figure 8:**
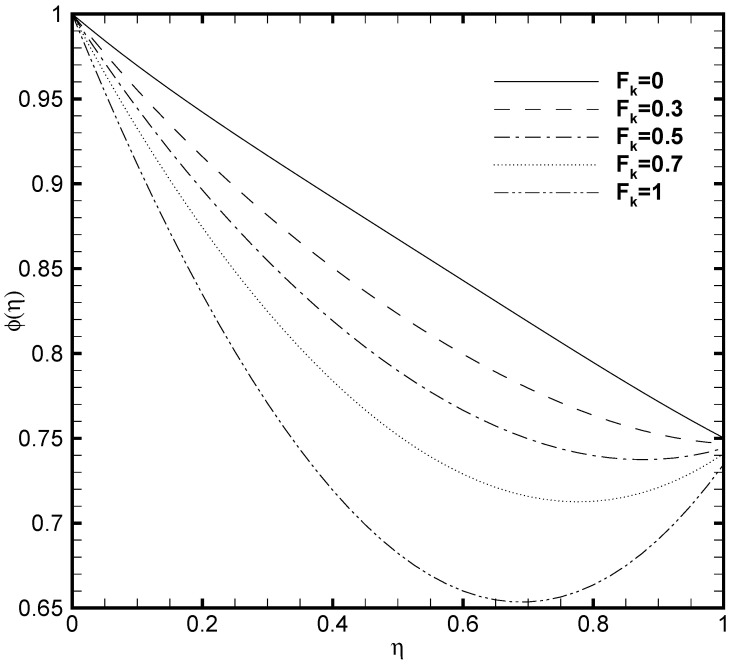
Nanoparticles concentration profile ϕ(η) for several values of Frank-Kamenetskii number $F_{k}$ when $\zeta =P=\kappa =Ha=Br=\delta_{\theta 2}=1$, $N_{b}=0.2$, $N_{t}=0.1$, β=0.05 and $\delta_{\theta 1}=0.5$.

Since the viscous dissipation effect is also considered for the present problem, which gives the ratio of viscous heat generation with external heating. Consequently, it is necessary to adequately observe the impact of Brinkmann number both on the temperature profile and nanoparticles concentration. In Figure 5 and Figure 6, the effect of Brinkmann number on the θ(η) and ϕ(η) are depicted, respectively. It is noted that as the value of Br evolves, the temperature profile increases while the nanoparticle volume fraction decreases. This is due to the increase in the Br value which cause the increase in the heat energy produced by the viscous dissipation effect. The influence of the Frank-Kamenetskii number ($F_{k}$) is described in Figure 7 and Figure 8, respectively. It is determined from Figure 7 that the dimensionless temperature profile elevates for the increasing values of $F_{k}$. The higher values of $F_{k}$ increases the heat generation caused by the exothermic reaction and it accelerates the convection mechanism within the channel. For $F_{k}=0$ then there is no exothermic reaction, so, θ(η) and ϕ(η) curves do not exhibit any variation. The nanoparticles volume fraction profile shows Figure 8 an opposite trend as compared to θ(η) for the increasing value of $F_{k}$. For greater value of $F_{k}$ the ϕ(η) decreases because the nanoparticles relocates from the hot region (upper plate that is, $\delta_{\theta 2}=1$) to the cold region (lower plate, i.e., $\delta_{\theta 1}=0.5$) caused by the effect of thermophoresis.

### 3.5. Effects of Temperature Constant ($\delta_{\theta 1}$), Brownian Diffusion ($N_{b}$) and Thermophoresis ($N_{t}$) Parameters

**Figure 9:**
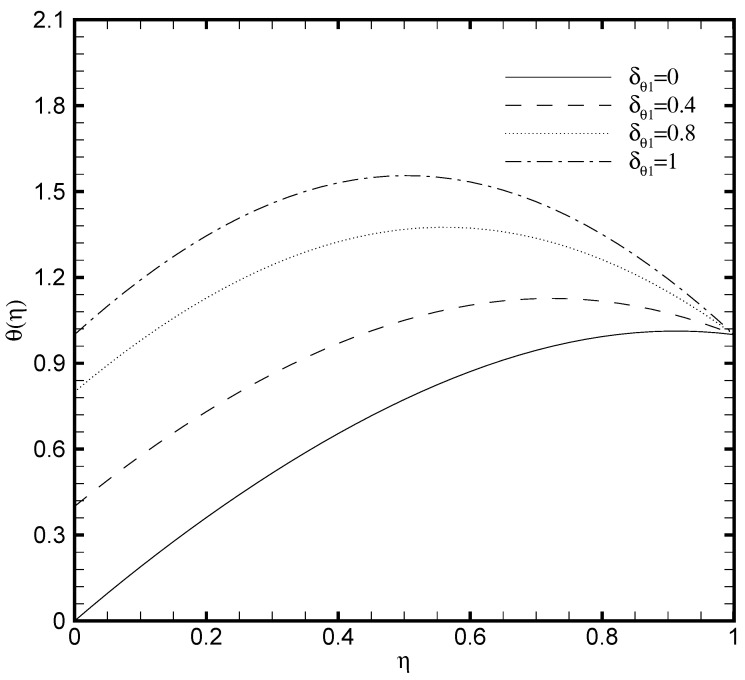
Temperature profile θ(η) for several values of a constant $\delta_{\theta 1}$ when $\zeta =P=\kappa =Ha=Br=\delta_{\theta 2}=F_{k}=1$, $N_{b}=0.2$, $N_{t}=0.1$, and β=0.05.

**Figure 10:**
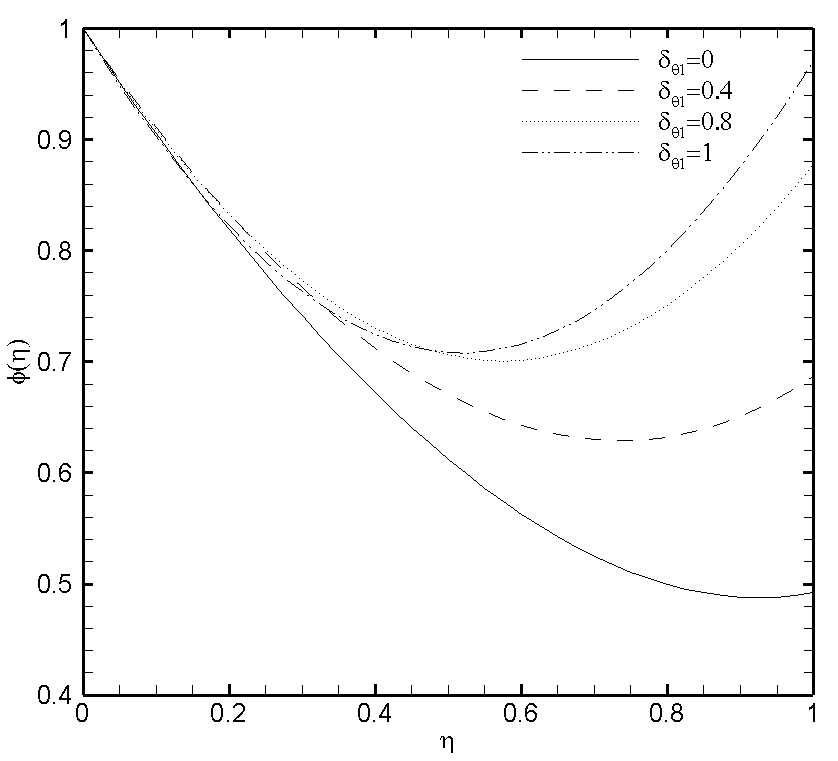
Nanoparticle volume fraction profile ϕ(η) for several values of a constant $\delta_{\theta 1}$ when $\zeta =P=\kappa =Ha=Br=\delta_{\theta 2}=F_{k}=1$, $N_{b}=0.2$, $N_{t}=0.1$, and β=0.05.

**Figure 11:**
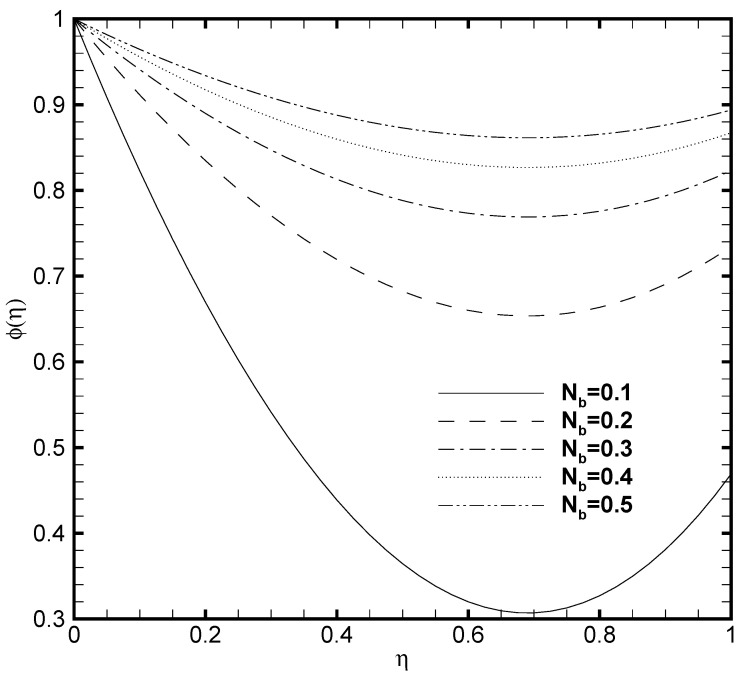
Nanoparticle volume fraction profile ϕ(η) for several values of Brownian motion parameter $N_{b}$ when $\zeta =P=\kappa =Ha=Br=\delta_{\theta 2}=F_{k}=1$, $N_{t}=0.1$, β=0.05 and $\delta_{\theta 1}=0.5$.

**Figure 12:**
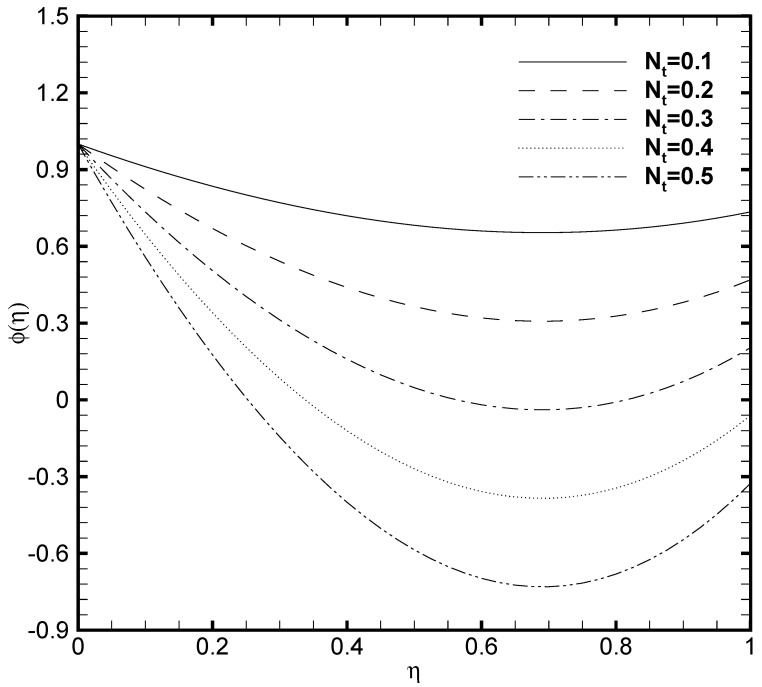
Nanoparticle volume fraction profile ϕ(η) for several values of thermophoresis parameter $N_{t}$ when $\zeta =P=\kappa =Ha=Br=\delta_{\theta 2}=F_{k}=1$, $N_{b}=0.2$, β=0.05 and $\delta_{\theta 1}=0.5$.

In the present work, the upper plate of the microchannel is fixed at a constant temperature value that is, $\delta_{\theta 2}=1$ while the temperature variates at the lower plate of the microchannel. Consequently, the profound effect of the lower wall temperature on the θ(η) and the ϕ(η) are displayed in Figure 9 and Figure 10. From Figure 9 it is seen that variation in temperature at the lower wall of microchannel exerts a noted impact on the temperature profile. The rise in the value of $\delta_{\theta 1}$ enhances the fluid temperature between the two walls. A similar behaviour is noted in Figure 10, where the ϕ(η) rises with the possible enhancement of the lower wall temperature and shows the dependence of nanoparticle volume fraction on $\delta_{\theta 1}$. It should be noted that the passive control nanoparticle concentration is employed at the upper wall while the active control model is adopted at the lower wall. Figure 11 and Figure 12 describe the potential effect of Brownian diffusion ($N_{b}$) and thermophoresis parameters ($N_{t}$) on the nanoparticle volume fraction. The ϕ(η) increases with the increase in the value of $N_{b}$ as displayed in Figure 11. While the observed increase in the $N_{t}$ significantly decreases the nanoparticle volume fraction as plotted in Figure 12. It is also observed from both figures that possible variation of nanoparticles concentration at the upper wall with the varying $N_{b}$ and $N_{t}$ is due to the passive boundary condition. This course also matches with the printed results of Kuznetsov and Nield [31].

### 3.6. Analysis of Physical Quantities

**Figure 13:**
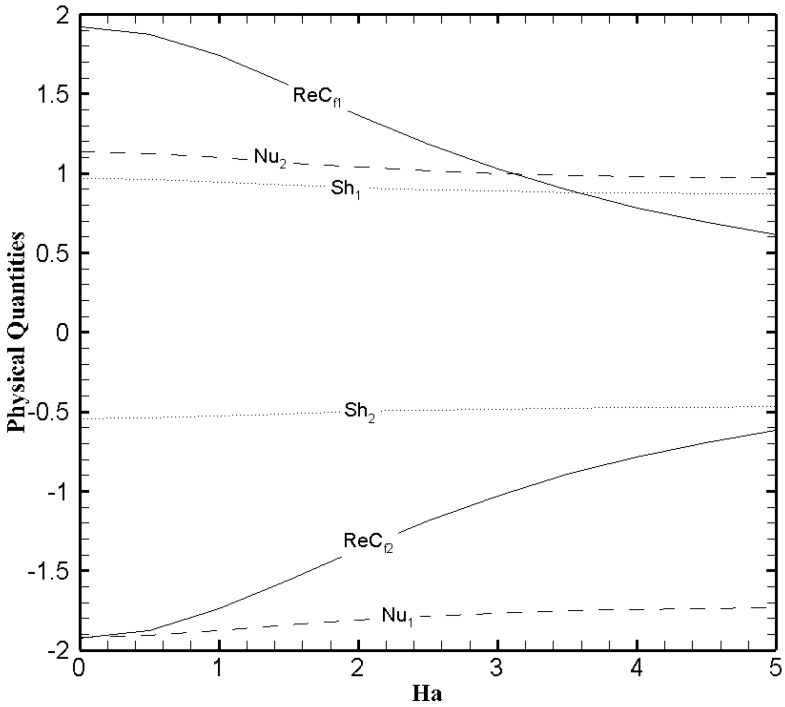
The physical quantities with Ha when $\zeta =P=\kappa =Br=\delta_{\theta 2}=F_{k}=1$, $N_{b}=0.2$, $N_{t}=0.1$, β=0.05 and $\delta_{\theta 1}=0.5$.

**Figure 14:**
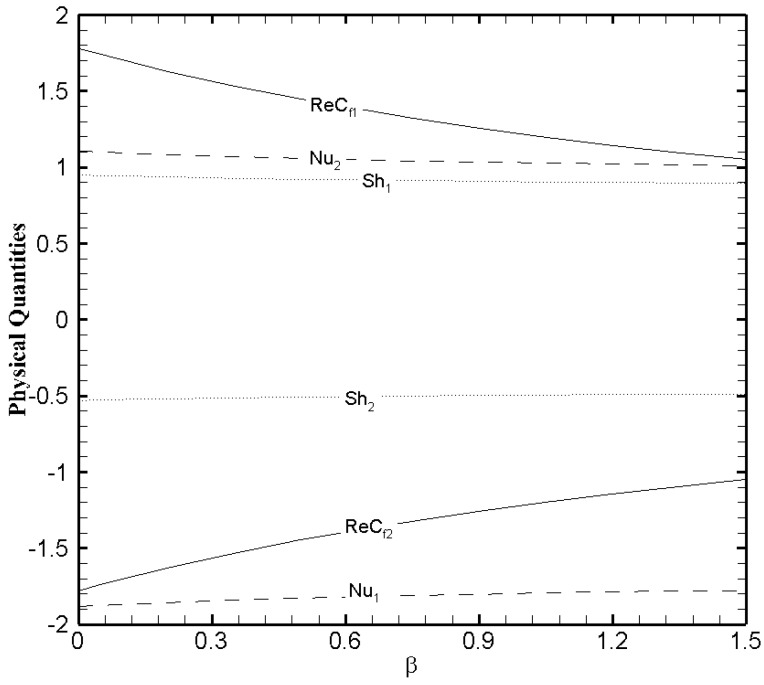
The physical quantities with β when $\zeta =P=\kappa =Ha=Br=\delta_{\theta 2}=F_{k}=1$, $N_{b}=0.2$, $N_{t}=0.1$ and $\delta_{\theta 1}=0.5$.

**Figure 15:**
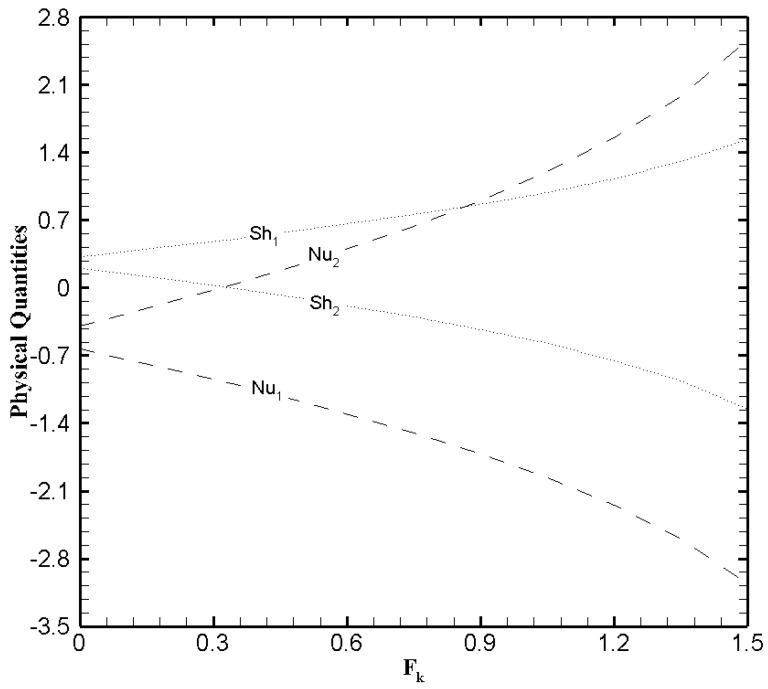
The physical quantities with $F_{k}$ when $\zeta =P=\kappa =Ha=Br=\delta_{\theta 2}=1$, $N_{b}=0.2$, $N_{t}=0.1$, β=0.05 and $\delta_{\theta 1}=0.5$.

To better comprehend the heat and mass transfer phenomenon in the microchannel the physical quantities that is, skin friction coefficient, Nusselt number, and Sherwood number must be explored. Figure 13, Figure 14 and Figure 15 show the variational trend of Ha, β and $F_{k}$ on the physical quantities. It can be perceived from Figure 13 that the skin friction decreases at the lower wall and increases at the upper wall as the value of Ha evolves. But the effect of Hartmann number on the Nusselt number and Sherwood number is negligible. The skin friction, Nusselt number and Sherwood number at both walls illustrate a similar trend with the slip parameter β (Figure 14) like Hartmann number. In Figure 15, the Nusselt number and Sherwood number is plotted against $F_{k}$. A decreasing trend in Nusselt number is observed on the lower wall while a gradual increase on the upper wall is noticed as the values of Frank-Kamenetskii number increase. In addition, an increase in Sherwood number is observed on the lower wall and decreases on the upper wall with the increase in the value of $F_{k}$. The effects of $F_{k}$ on the skin friction is negligible.

## 4. Conclusions

The mathematical modeling and physical analysis have been done for the fully developed steady MHD nanofluid flow through the microchannel in the presence of EDL effects and exothermic chemical reactions with slip boundary conditions. Buongiorno’s model [29] has been properly employed to formate the mathematical model for the nanofluids. The dimensional non-linear system of fundamental equations has been converted to non-dimensional non-linear differential equations via similarity transformation. Then the numerical solutions are obtained and physical analysis has been done by plotting the graphical figures of various physical parameters. The key findings of this work are as follows:

1. The exact solutions are obtained for both the electric potential and velocity field with the corresponding slip conditions.
2. The slip parameter exercises a significant influence on the velocity field but the effectiveness of the boundary slip gets negligible when the value of zeta potential is large enough. This analysis supports the published findings by You and Guo [25].
3. The temperature profile greatly elevates due to the heat generation caused by the exothermic chemical reaction. While for more elevated Frank-Kamenetskii number the nanoparticle volume fraction decreases.
4. Frank-Kamenetskii number display a noticeable effect on the Nusselt number and Sherwood number. While Ha and β exert a significant effect on skin friction.

## Data Availability

The data presented in this study are available on request from the corresponding author. The data include the MATLAB code to solve the governing equations and the figures. Not making them public is because governing equations can easily be solved by any numerical method or build in MATLAB functions.

## Abbreviations

**Nomenclature**
Br	Brinkman number
$C_{1}$	nano-particle volume fraction
$C_{0}$	reference nano-particle volume fraction
$C_{f1},C_{f2}$	local skin friction coefficients
${\left(c_{p}\right)}_{f},{\left(c_{p}\right)}_{p}$	specific heat capacity of fluid and nanoparticles
$Nu_{1},Nu_{2}$	local Nusselt numbers
$Sh_{1},Sh_{2}$	local Sherwood numbers
$q_{T1},q_{T2}$	Heat flux on the microchannel walls
$q_{C1},q_{C2}$	mass fluxes on the microchannel walls
$D_{B}$	Brownian diffusion coefficient
$D_{T}$	thermophoretic diffusion coefficient
$k_{nf}$	thermal conductivity of the fluid
*P*	non-dimensional pressure gradient parameter
$T_{1},T_{2}$	temperatures of nanofluid on microchannel walls
$T_{0}$	reference temperature
$U_{m}$	average velocity of the fluid
Ha	Hartmann number
$F_{k}$	Frank-Kamenetskii number
Ec	Eckert number
Pr	Prandtl number
*E*	activation energy
$E_{e}$	electric field strength
*e*	charge of proton
$E_{x}$	electric field in x-direction
*F*	electrical body force from uniform electromagnetic field
$k_{b}$	Boltzmann constant
$n_{0}$	the bulk ionic concentration
$N_{b}$	Brownian motion parameter
$N_{t}$	thermophoresis parameters
Re	Reynolds number
$\hat{z}$	the valences of ions
**Greek Symbols**	
α	nanofluid’s thermal diffusivity
ϵ	ration of dielectric constants of the medium;
$\epsilon_{0}$	permittivity of vacuum
$\epsilon_{r}$	dielectric constant of the medium
μ	dynamic viscosity of the fluid
Ψ	non-dimensional electrostatic potential
${\left(\rho_{n}\right)}_{f}$	nanofluid density
${\left(\rho_{n}\right)}_{p}$	nanoparticles density
$\rho_{e}$	charge density
σ	electrical conductivity of fluid
$\beta^{*}$	dimensional slip parameter
β	dimensionless slip parameter
κ	electro-osmotic parameter
θ	non-dimensional temperature parameter
ϕ	non-dimensional nanoparticles volume fraction
$\delta_{\theta 1},\delta_{\theta 2}$	constants on the microchannel walls
ζ	non-dimensional zeta potential
$\bar{\zeta }$	zeta potential
$\tau_{w1},\tau_{w2}$	wall shear stress on the microchannel
